# N-BiC: A method for multi-component and symptom biclustering of structural MRI data: Application to schizophrenia

**DOI:** 10.1109/TBME.2019.2908815

**Published:** 2019-04-01

**Authors:** Md Abdur Rahaman, Jessica A. Turner, Cota Navin Gupta, Srinivas Rachakonda, Jiayu Chen, Jingyu Liu, Theo G. M. van Erp, Steven Potkin, Judith Ford, Daniel Mathalon, Hyo Jong Lee, Wenhao Jiang, Bryon A. Mueller, Ole Andreassen, Ingrid Agartz, Scott R. Sponheim, Andrew R. Mayer, Julia Stephen, Rex E. Jung, Jose Canive, Juan Bustillo, Vince D. Calhoun

**Keywords:** multi-component and symptom biclustering, schizophrenia, structural MRI; N-BiC: N-way biclustering, SYMBiCs: Symptom bicluster, subtypes, independent component analysis

## Abstract

**Objective:**

We propose and develop a novel biclustering (N-BiC) approach for performing N-way biclustering of neuroimaging data. Our approach is applicable to an arbitrary number of features from both imaging and behavioral data (e.g., symptoms). We applied it to structural MRI data from patients with schizophrenia.

**Methods:**

It uses a source-based morphometry approach (i.e., independent component analysis (ICA) of gray matter segmentation maps) to decompose the data into a set of spatial maps, each of which includes regions that covary among individuals. Then the loading parameters for components of interest are entered to an exhaustive search, which incorporates a modified depth-first search (DFS) technique to carry out the biclustering, with the goal of obtaining submatrices where the selected rows (individuals) show homogeneity in their expressions of selected columns (components) and vice versa.

**Results:**

Findings demonstrate multiple biclusters have an evident association with distinct brain networks for the different types of symptoms in schizophrenia. The study identifies two components: inferior temporal gyrus (16) and brainstem (7), which are related to positive (distortion/excess of normal function) and negative (diminution/loss of normal function) symptoms in schizophrenia respectively.

**Conclusion:**

N-BiC is a data-driven method of biclustering MRI data that can exhaustively explore relationships/substructures from a dataset without any prior information with a higher degree of robustness than earlier biclustering applications.

**Significance:**

The use of such approaches is important to investigate the underlying biological substrates of mental illness by grouping patients into homogeneous subjects as the schizophrenia diagnosis is known to be relatively nonspecific and heterogeneous.

## I. Introduction

BICLUSTERING is a data mining technique that is a powerful tool for high dimensional biological data analysis. It has been successfully applied to bioinformatics, primarily in microarray gene expression data for discovering local patterns, yet it is a comparatively new approach as applied to magnetic resonance imaging (MRI) analysis. It has been applied to structural neuroimaging data, but only on a single pair of features. Biclustering is more generally a data mining technique that allows simultaneous clustering of the rows and columns of a matrix; thus, biclusters are a subset of rows that exhibit homogenous values across a subset of columns and vice-versa. In gene expression data, a bicluster is usually a subset of genes that exhibit compatible expression patterns over a subset of conditions [1]. For neuroimaging entities, a bicluster is a subset of subjects which show similar patterns across a subset of variables/features [2]. More specifically in our analysis, a bicluster is a subset of subjects who are relatively homogenous across a subset of imaging patterns. Previous neuroimaging studies of schizophrenia (SZ) have suggested that clustering could help identify schizophrenia subtyping/groupings and characterizing their neural correlates [3–6]. In an earlier study, we performed biclustering on two neuroimaging components selected based on prior information [3], and we identified groups of subjects with disjunctions of symptoms (e.g., severe in one dimension and not in the other vs severe in both).

A major challenge for clinical and biological research trying to elucidate causes of schizophrenia is the heterogeneity of the illness. The wide range of clinical and cognitive symptoms within this disease restricts the isolation of specific neural systems or functional neural markers of the disorder [7]. To resolve the issue of variability in SZ, researchers have attempted to define subtypes based on clinical characteristics. But such an approach has also been criticized [8, 9] because of a lack of strong theoretical background and the relative absence of neurobiological correlates [10–13], as well as the temporal instability of clinical symptoms and their corresponding subtypes [4]. Subtypes suggested by the diagnostic systems tend to be unstable and change over a short time period [4]. Hence, it has been suggested that the diverse clinical syndromes of schizophrenia should be subgrouped based on distinct symptom profiles of the disorder [14, 15]. As a result, researchers are focusing on reliable and stable genetic and neuroimaging data rather than clinical features in schizophrenia [16]. Two widely used approaches for analyzing structural MRI data include voxel-based morphometry (VBM) [17–19] and its multivariate extension, source-based morphometry (SBM) [20–22]. VBM is a univariate analysis that allows examination of brain changes in a voxel by voxel model, instead of focusing on spatial patterns across voxels [23]. SBM is a multivariate analysis implemented by applying independent component analysis (ICA) to gray matter maps and their associated loading parameters and identifying spatial regions that covary across individuals. Multivariate analysis such as SBM can increase sensitivity to distributed effects, providing a stronger prospect of automatically diagnosing an individual and their association with certain clinical subgroups [24]. While SBM has been applied to structural imaging more broadly, to our knowledge no MRI study has attempted to bicluster individuals based on multiple combinations of neuroimaging features and symptom scores.

The fundamental goal of our study is to develop a novel biclustering method to identify biclusters that include subgroups of individuals or neuro features from MRI data as along with symptom data. This is the first application of biclustering to multiple neuroimaging and symptom features with no prior assumptions about the features, dataset or imposed external objective function. The proposed algorithm can be applied to any MRI dataset irrespective of size or dimension (given that the data set satisfies the minimum subjects and component requirements of the algorithm). It identifies homogeneous subsets of individuals similarly expressed across a neuroimaging feature vector or subsets of identical neuroimaging features that maintain homogeneity within a group of individuals. N-BiC groups subjects and their neural features simultaneously from a given collection of subjects and features by maximizing homogeneity across both dimensions. The algorithm creates a list of biclusters where each BiC consists of a subset of subjects and features. We apply biclustering to all possible subsets of imaging features using a depth-first search (DFS) technique [25]. First, we apply the method on simulation data and visualize the results to demonstrate the approach and justify the parameter choices. Next, we apply it to an aggregated MRI dataset of patients with schizophrenia.

A focal aim of our study is to explore the relationship between symptom scores and the features directly by biclustering them together, rather than using that information just for post hoc analysis. The goal here is to extract biclusters that help establish subgroupings or endophenotypes. In this work, we focus on neuroimaging features and symptom scores. For neuroimaging features, we include the SBM loading parameters. For symptoms, we include positive and negative syndrome scale (PANSS) scores. We used our algorithm to identify biclusters from the resulting loading parameters and symptom scores and quantify the relationship between the subgroups (biclusters or BiCs) and the symptom scores. We applied the algorithm to a dataset consisting of 382 schizophrenic subjects to aim at identifying structurally distributed signatures that directly differentiate patients. The results identify submatrices (BiCs) of the given loading matrix with a group of subjects showing homogenous neurological signatures across a set of spatial components. Biclusters that included more than one symptom were labeled SYMBiC.

## II. Methods

**Fig. 1.**
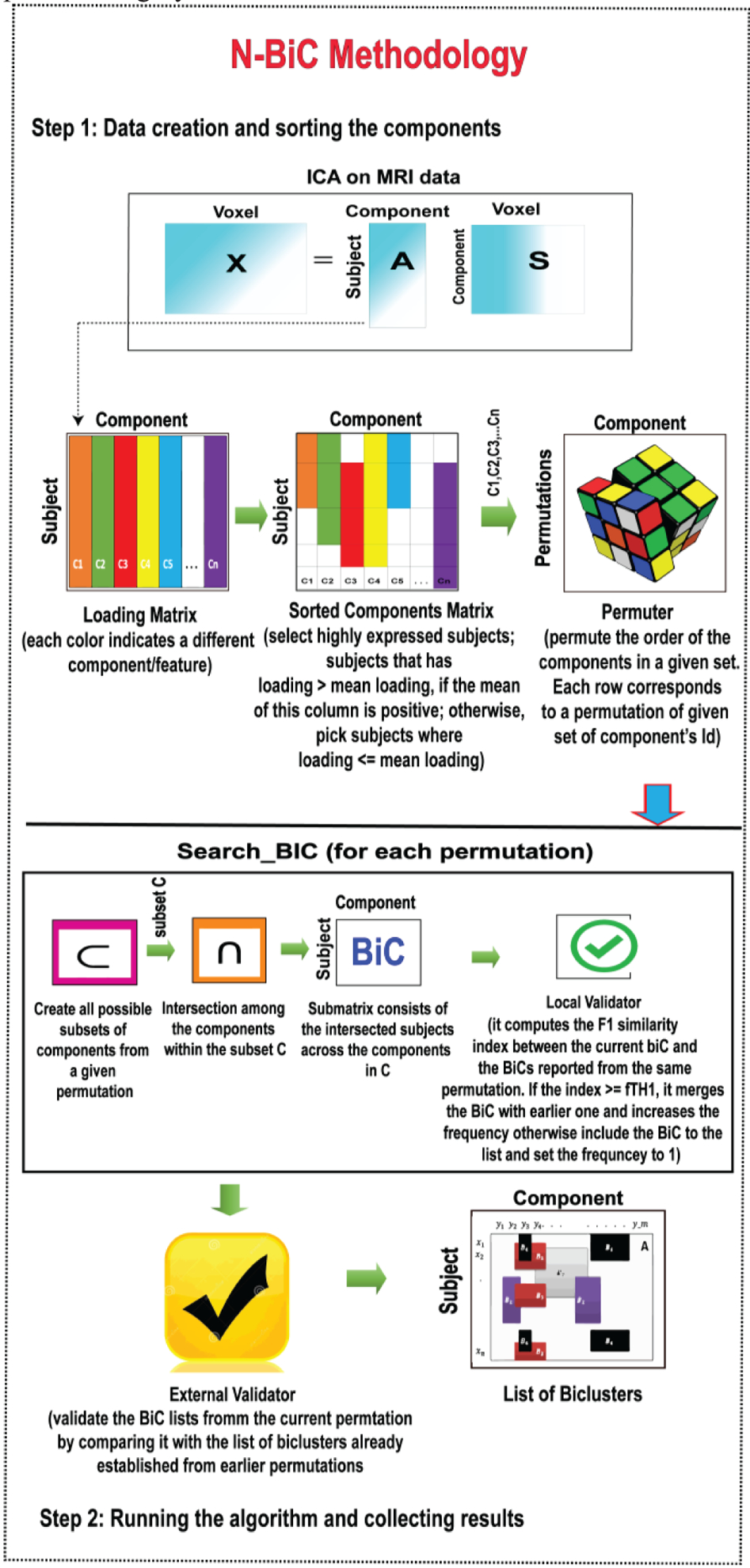
Block diagram demonstrating the methodology

We described the analysis in two basic steps. In the first step, we create and sort the components using the method described herein. Then, we assign an arbitrary numeric ID to each of the sorted components (a subset of subjects) according to their order in the loading matrix. For example, if we have m components in the loading matrix [n (number of subjects) by m], then we also have m sorted components (SCOMPs) labeled 1 to m. In step 2, we create all possible permutations of the IDs of SCOMPs and send them to a Search_BIC function to determine the biclusters. By checking all SCOMP permutations, we are progressively looking at the different sequence of components to make our analysis invariant to the order in which we are evaluating the components. The Search_BiC function first evaluates all possible subsets of a given set of IDs using the selected parameters. At each iteration, Search_BiC evaluates the intersection among the SCOMPs within the subset and forms a submatrix of the intersecting SCOMPs and the subset of subjects after intersection (SUB) (each SCOMP is a subset of subjects); this submatrix is a Bicluster or BiC. If we are intersecting SCOMP s1 s2, and s3, then the BiC consists of components s1, s2, s3 and subjects (number of subjects in SUB). Then, the BiC is sent to a local validator where it is checked for size constraint and a similarity index to compare the overlap between the biclusters from the same permutation. The process is performed for all subsets produced by a single permutation. The Search_BIC function on each permutation returns a temporary list of biclusters that has been passed through a global stability checker, which performs the validation of the temporary lists of biclusters and creates a final list of stable biclusters. (We provide more details on the stability checker in paragraph B.) A block diagram demonstrating the various steps of our methodology is shown in figure 1.

### A. Image Processing

For data acquisition, scanning sites used 1.5 and 3 T scanners of various models, collecting T1-weighted images using sagittal or axial orientation and MPRAGE sequences. We followed the preprocessing pipelines used in Ref. [21, 22, 26]. We used preprocessed data from Ref. [3]. Images were spatially normalized to the 152 average T1 Montreal Neurological Institute template using a 12-parameter affine model followed by a nonlinear model; resliced to 2 × 2 × 2 mm; and segmented into gray, white, and CSF images using the unified segmentation algorithm from SPM5 [17, 21, 22]. Full width half maximum Gaussian kernel (FWHM) of 10 mm was used to smooth the images as suggested in Ref. [27]. Subject outlier detection was performed using a Pearson correlation, which compared the degree to which subjects are like the average smoothed GM map. If we found an outlier, we visually checked the subject, corrected and re-segmented if possible. In a few cases, we removed subjects where it was not possible to fix the problem. For details about the image processing, it is advised to check with the reference papers [3, 20, 28]. After preprocessing, spatial ICA was used on the gray matter images to estimate spatial components and their loading matrix, reflecting spatial patterns of gray matter covariation across individuals.

The number of components was set to 30 and we used ICASSO (30 runs followed by a selection of the most central run) to ensure the stability of the components. The dataset was decomposed into 30 SBM components resulting in a 382-by-30 loading matrix. We selected nine components from the 30 that showed a significant effect of diagnosis in a previous study by Gupta et al. [20]. The components are comprised of multiple cortical, subcortical, and cerebellar regions. Larger loading parameters for an individual or group indicate that the spatial pattern is more strongly weighted in the data for that individual or group [20]. The loading coefficients matrix and PANSS scores are provided as input into our algorithm.

### B. Algorithm

**Fig. 2.**
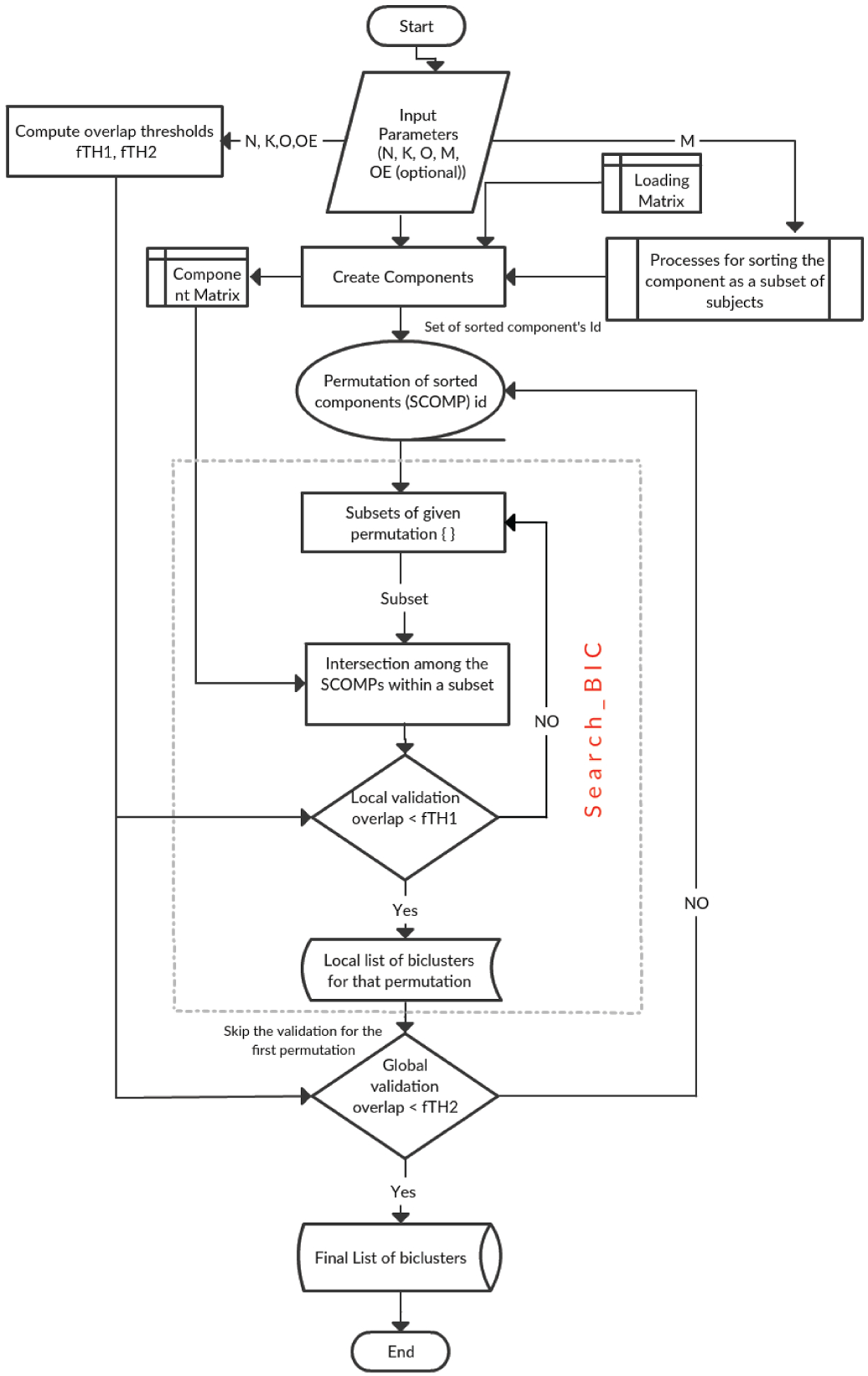
Flowchart of the implemented N-BiC algorithm

The algorithm uses a modified DFS strategy [25] for exploring a data matrix by evaluating all possible subsets of a given set of numbers where each number could potentially represent a distinct column/row of that matrix depending on the problem definition. In our study, these numbers mostly indicate neural variables (i.e., components/features). The steps of our algorithm are depicted in figure 2 by a flowchart. The input parameters for the algorithm are as follows:

L: Data matrix to be biclustered
N: Minimum number of subjects in a bicluster
K: Minimum number of components/features in a bicluster
O: Allowed percentage of overlap between the biclusters
M: Preferred method for sorting the components
OE: Allowed percentage of overlap between the biclusters from different permutations (optional)

If not specified, O is going to be used for both overlap thresholds.

### C. Sorting the Components/Features

We proposed four methods for sorting the components. The basic idea of sorting is to select the subjects with higher loading values for a feature. In other words, we select only those subjects where the feature is highly expressed. The methods compare the subject’s contribution (loading) from a component with the average loading value of that feature to include this subject into the subset. The subjects are annotated with distinct numeric labels. For instance, if we have a loading matrix of n × m dimensions, then the subject labels are 1 to n and the components labels are 1 to m consistent with their order. Sorting decomposes the matrix into m subsets of subjects where each subset represents a sorted component (SCOMP) and every SCOMP is a collection of integers ranging from 1 to n (number of subjects) with an arbitrary size. We created a simulated dataset to check the performance of those methods. Then, we analyzed the extracted results by these methods for two evaluation indices (mean square residue, consensus score) to determine the optimal method. The methods are as follows:

*Method 1:* Positive and Negative: Calculate means for subjects with positive and negative loading separately. Then, select subjects from the cohort that satisfy the following equations, *loading* ≥ *positive mean*, *loading* ≤ *negative mean*

*Method 2:* Positive and negative quartiles: Select individuals from the upper quartile of positive loading and the lower quartile of negative loading.

*Method 3:* Absolute value: Select individuals who have a loading parameter greater or equal to the mean loadings of that column (component) irrespective of their sign. Take subjects where |*loading*| ≥ |*meanloadings*|

*Method 4:* Positive or negative: Select subjects with only positive or only negative loading based on the overall mean loading of that column. That is, if the mean loading of the feature is positive, then we select individuals with extremely positive loading parameters; otherwise, select individuals with extremely negative loadings. The following equations define extremely positive and negative loadings,

*Ex. positive, loading* ≥ *mean loadings*,

*Ex. negative, loading* < *mean loadings*.

### D. Performance Comparison Sorting Methods (Simulation-1)

To determine the best method, a simulated dataset consisting of 400 subjects and 10 features was developed. We used a normal distribution with different means to generate simulated loading values within [−1,1]. These four methods were applied to the customized dataset to sort the features. We also developed a set of simulated biclusters with size constraints (35, 3) (minimum number of subjects, the minimum number of components) using ‘randn’ functions from MATLAB [Table I]. We selected ≥ 35 subjects ranging from 1–400 and 3 random components ranging from 1–10 since we have 400 subjects and 10 features in our simulated loading matrix. Then, we embedded these into the data matrix. The embedding is straightforward; we increased the loading value of those cells of a data matrix that belong to any of the simulated biclusters. We adjusted the expression (loading) values in such a way that it always remains higher than the positive mean of that column (component), which increases the likelihood of that subject being included in the corresponding sorted component (SCOMP). Since the fundamental measure of these four methods is the mean loading of a feature, this remodeling of the data matrix assigns an equal likelihood of being included in corresponding sorted components to all the cells that belong to the list of simulated biclusters. As a result, these four blocks of cells (4 BiCs) behave like four intrinsic biclusters in the dataset. Finally, we implemented N-BiC on this customized data matrix for extracting biclusters (Table I).

**Fig. 3.**
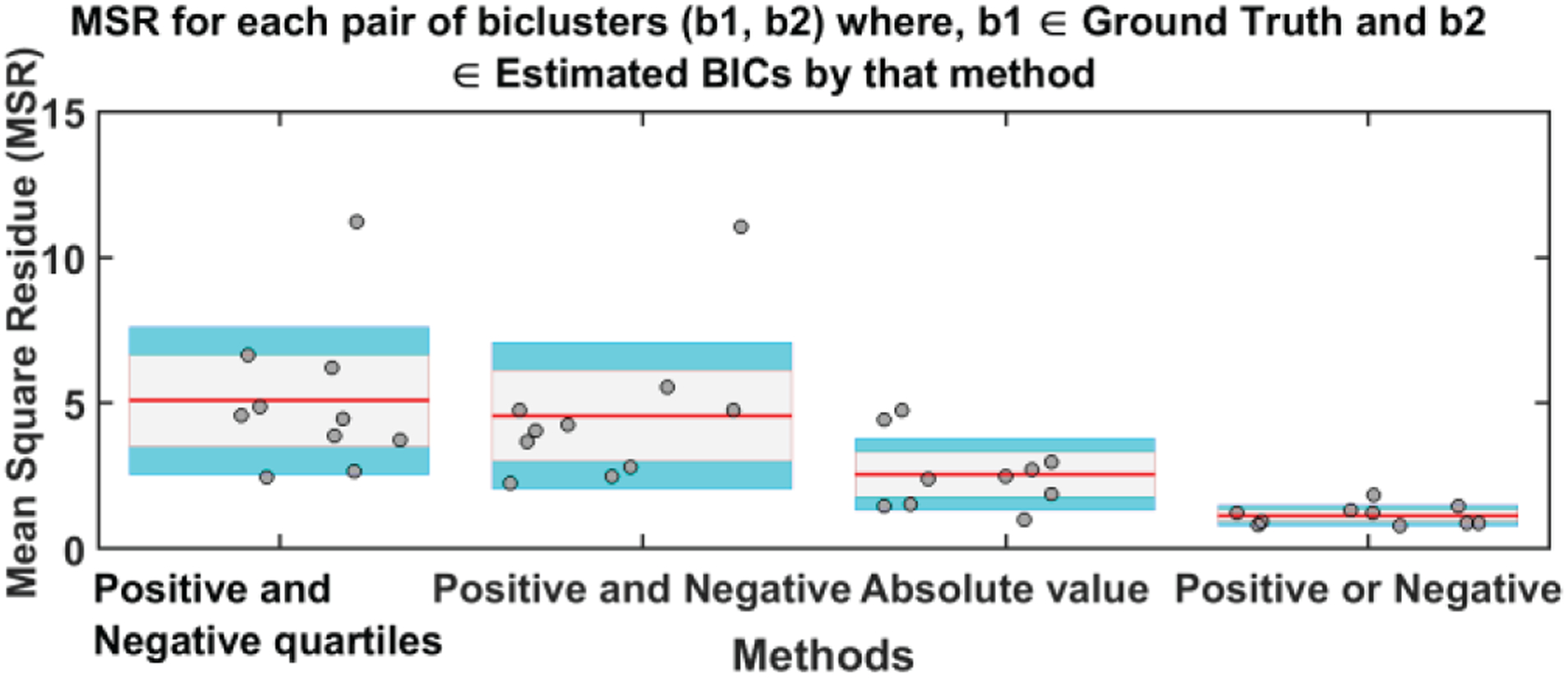
Mean MSR (red line on each bar) and standard deviation of the values for each method. In this case, lower values are better; again, the positive or negative approach showed the best performance. The gray area represents the standard error of the mean (mean ± SEM) and the blue area is 1 standard deviation (mean ± SD) from the mean.

The resulting set of biclusters was evaluated using different metrics to quantify the strength, stability, and coherence of biclusters. We computed the mean square residue (MSR), F1 similarity index and a consensus score (cSCORE) to identify the best method [29, 30]. The MSR is a measure of coherence of a given bicluster. The lower the MSR, the stronger the coherence shown by the bicluster [30]. We calculated the MSR for each BiCs and then averaged all BiC MSR values for a method. Figure 3 depicts the average MSR and the standard deviation of MSR values of each pair of biclusters where one is taken from the estimated BiC and another from the set of ground truth BiCs for every method. As we show, the positive or negative method (method 4) was found to be the best approach (i.e., the one exhibiting the lowest MSR value). Since the embedding of the biclusters is consistent for all four methods, there is no clear bias of embedding on the biclustering results. Since we have the true set of biclusters, we also evaluated the *consensus score (cSCORE)* to assess consensus between the set of extracted biclusters and the set of true biclusters [29]. We also computed the FABIA (factor analysis for bicluster acquisition) to quantify the consensus score using the F1 index which is a combination of sensitivity and specificity of a pair of biclusters [31, 32]. Based on the consensus score calculation, we computed the F1 index for each pair of biclusters and compared each bicluster from the true set with each bicluster extracted by a specific method [29]. To assemble a set of biclusters into a combined set of BiCs, we evaluated the sensitivity of each true bicluster compared to the estimated BiCs. When the sensitivity between BiCs was high, then we merged two BiCs together, resulting in a single larger BiC. Since the consensus score is the sum of the F1 index divided by the number of biclusters in the combined set, a higher cSCORE is obtained if the number of BiCs in the combined set is lower; this means the two sets of biclusters are very similar [29]. The maximum consensus score is 1 for two identical sets of biclusters. We present the consensus score bar graph and F1 similarity index in figure 4(a) and 4(b) respectively; the maximum cSCORE is associated with the positive or negative method (Method 4). Note that the value is not particularly large because we are comparing to two differently sized sets of biclusters (i.e., the size of the true set is four, whereas the extracted set contains at least 10 BiCs). For a similarity index, a higher index indicates higher similarity and thus better estimation, and the positive and negative method best performed for all four biclusters.

### E. Validator and Thresholds

For validating the biclustering results, we used a stability-based validation consisting of five consecutive steps. A high-level description of these five steps is given below:

*Step 1.* Obtain biclusters for the initial set of components

*Step 2.* Permute the given set

*Step 3.* Obtain biclusters for all permutations

*Step 4.* Compute the summary metrics on the bicluster solution using the F1 index

*Step 5.* Average the external indices.

Using the N, K, and O, the approach calculates two F1 similarity index thresholds (fTH1 and fTH2), which will be required by the local validator and global stability checker respectively. These thresholds control the similarity/overlap between the BiCs; fTH1 controls the internal overlaps with the biclusters from the same permutations and fTH2 restricts overlaps with biclusters from earlier permutations. For the similarity measure, we used the F1 index known as a dice index, a harmonic mean of precision and recall [31, 32]. The F1 index for two arbitrary biclusters A (estimated) and B (ground truth) is,

F1(A,B)=2|A∩B|/|A|+|B|

|A∩B| = Intersection between BiC A and B ; S_A∩B_ * F_A∩B_

|A| = Size of A; i. e. the number of subjects × components

|B| = Size of B; i. e. the number of subjects × components

Precision = ratio of relevant subjects selected to a number of features selected, |A ∩ B| / |B|

Recall = ratio of relevant features selected to a number of relevant subjects available, |A ∩ B| / |A|

According to the F1 index equation, fTH1 and fTH2 are defined as,

$$\text{fTH}1\geq \frac{2\times \frac{O\times N\times K}{100}}{N\times K+N\times K}$$

After simplification, $\text{fTH}1\geq \frac{O}{100}$ Similarly,

$$\text{fTH}2\geq \frac{\text{O}\;\text{or}\;\text{OE}}{100}$$

These are the minimum values for the thresholds since these are calculated using a minimum number of subjects, components and allowed overlap. However, the user can calibrate according to their studies and expectations.

### F. Searching the Biclusters

The first step is to sort components by a method specified by M from the loading matrix using the required variables and thresholds. As we mentioned earlier, the permuter computes all possible permutations of the IDs of sorted components (SCOMPs) within the component matrix. The permuter sends one permutation to the Search_BiC function in each iteration for all permutations in the permutation matrix. Initially, the function lists all possible subsets of the given permutation. In each iteration, it picks a subset and identifies the intersection among the SCOMPs (set of subjects) whose IDs are given in that subset. The intersection results in a set of subjects and we have the subset of SCOMP’s IDs and, after checking for size constraints (N, K), this forms the bicluster – a subset of subjects expressed homogenously across a subset of components. After that, the submatrix is sent to a local validator, which checks the overlap with biclusters from that permutation and uses fTH1 to verify the uniqueness. The processing is continued for all subsets produced from a given permutation. Then, the extracted biclusters are stored in a final list of biclusters. For the first permutation, there is no global stability checking since there are no biclusters from any earlier permutations. From the second permutation, the list of estimated biclusters from that permutation is checked for global stability by using fTH2 before being stored in the final list of BiCs. We will discuss these thresholds and the checker in the next paragraph. Depending on the decision from the global validator, the BiCs within the list is merged with an earlier BiC and increase the frequency or are introduced to the main list with a frequency 1.

## III. Results

### A. Results of performance comparison Indices (Simulation-1)

**Fig. 4(a).**
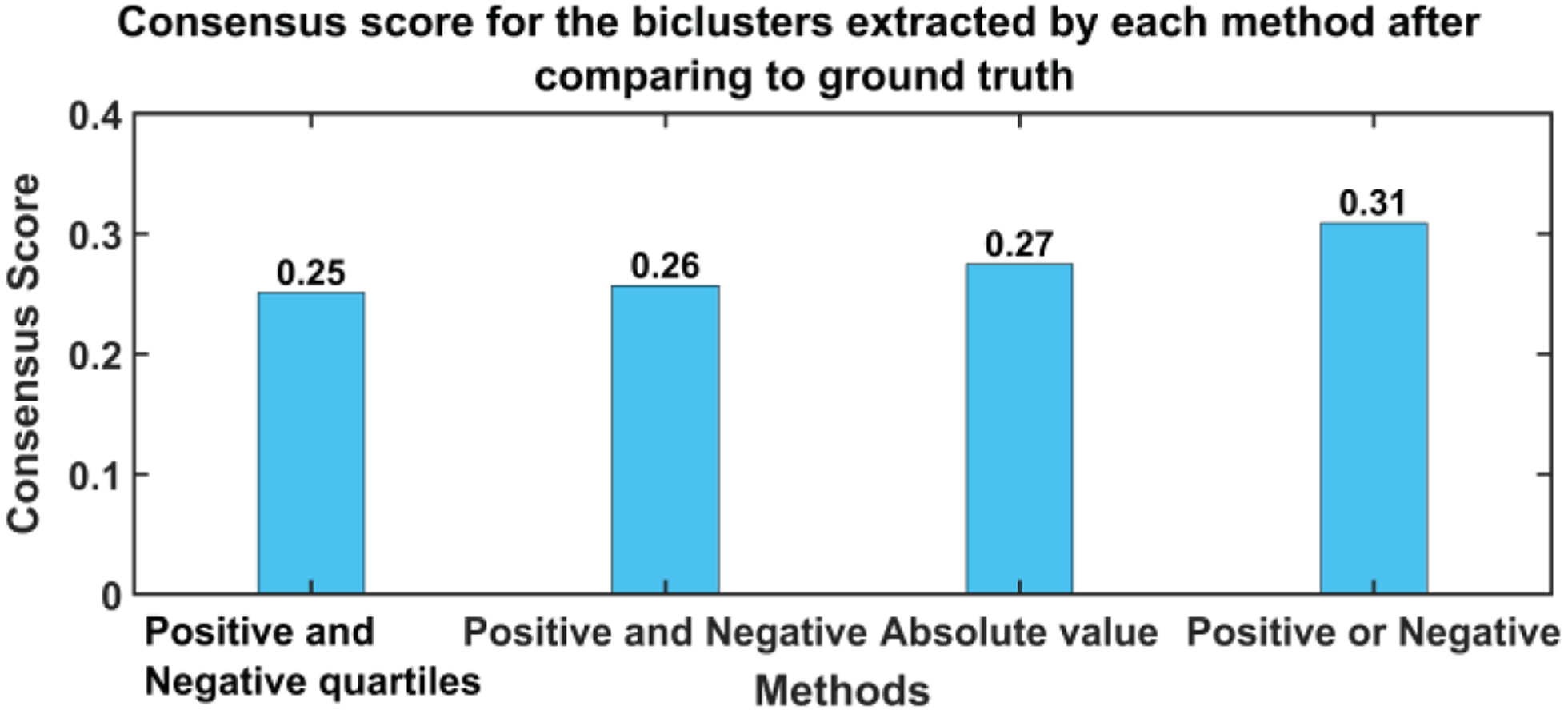
Consensus Score for all methods used for sorting the features, from one run, and thus does not include a confidence interval. A higher value indicates better performance.

**Fig. 4(b).**
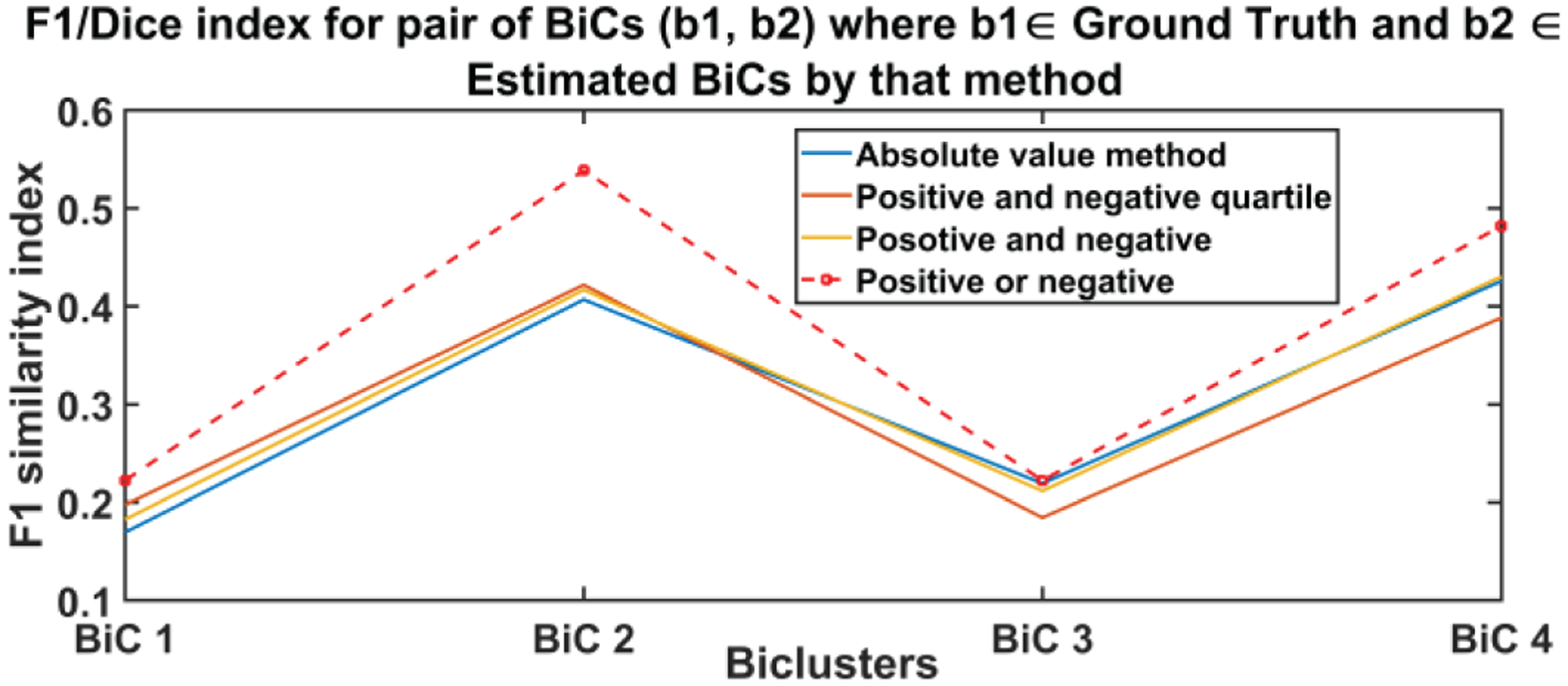
F1 similarity index between estimated and ground truth biclusters. A higher value indicates more similarity and thus a better estimation. Positive or negative method (red dotted line) outperformed the other three methods at all BiCs.

### B. Apply N-BiC On Toy Data (Simulation-2)

We ran our algorithms on both the simulated and real datasets. First, we developed a small simulated data set to demonstrate the basic functionality of N-BiC. The simulation data set consisted of 40 subjects and 10 components and three biclusters. The loading values and the biclusters are generated by the method we described in simulation-1. This time, we created a 40 by 10 data matrix and the bicluster has more than 11 random subjects with an interval 1–40 and at least 3 randomized features with an interval of 1–10 (Table II). Finally, we analyzed this data with the N-BiC algorithm.

**Fig. 5.**
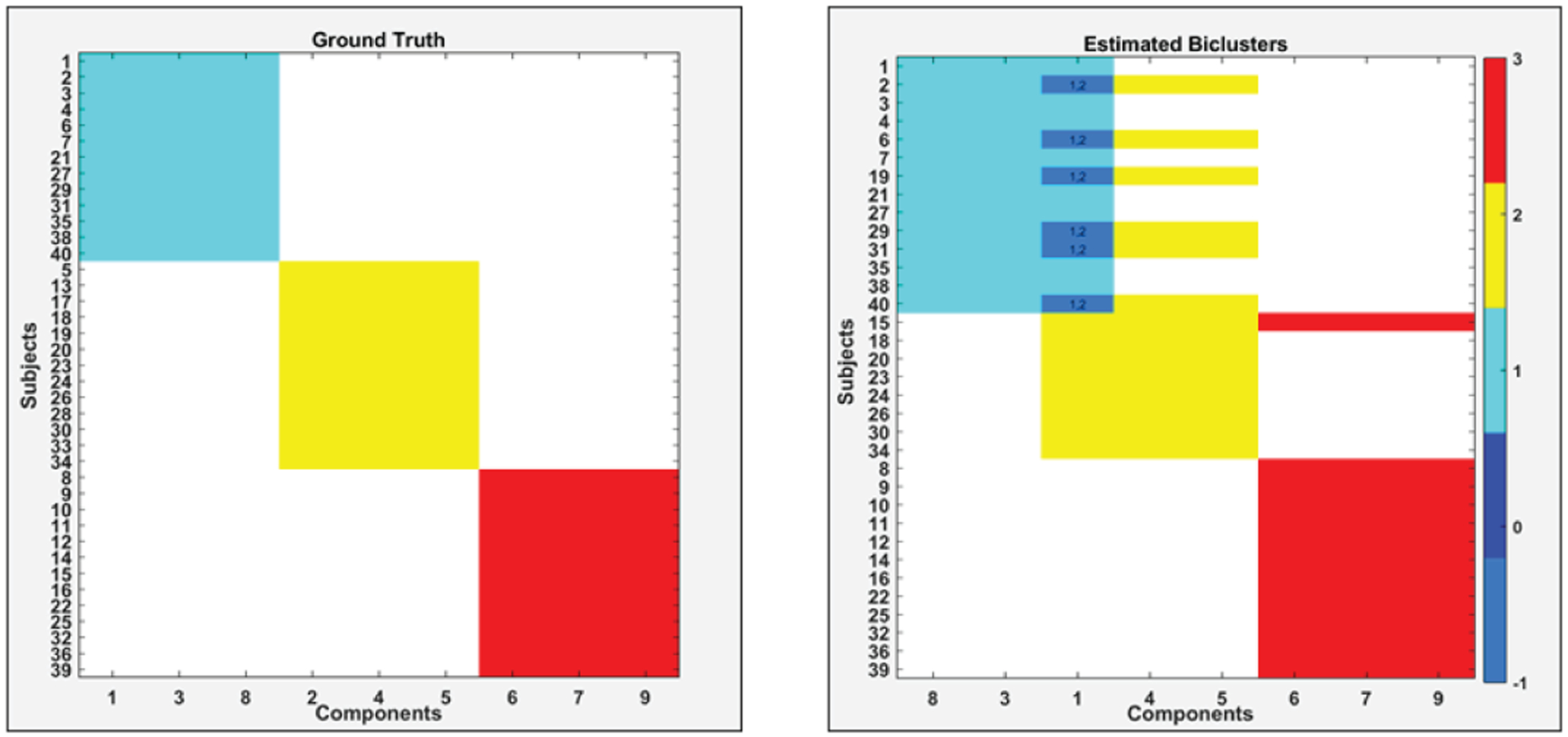
Comparison between ground truth (left) and estimated biclusters (right) from the simulated dataset-2 by using the N-BiC algorithm. In each case, components are shown on the x-axis and subjects in each cluster on the y-axis. Color bar on the right side indicates assigned color for different biclusters label.

Fig. 5 depicts the comparison between the ground truth and estimated biclusters by N-BiC methodology; subject and component bicluster identity are shown in different colors. Although in the ground truth there is no overlap in subjects, the estimated set shows a few that have overlapping subjects (e.g., component 1 and a small subset of subjects are common in both biclusters 1 and 2).

Generally, the estimated biclusters are largely similar pairwise to the ground truth, except for the overlapping subjects; one was wrongly assigned (subject 19 assigned to the blue cluster rather than the ground truth assignment of the yellow bicluster) and exchanging one component for another in the yellow bicluster.

### C. Apply N-BiC On Real Dataset

**Fig. 6.**
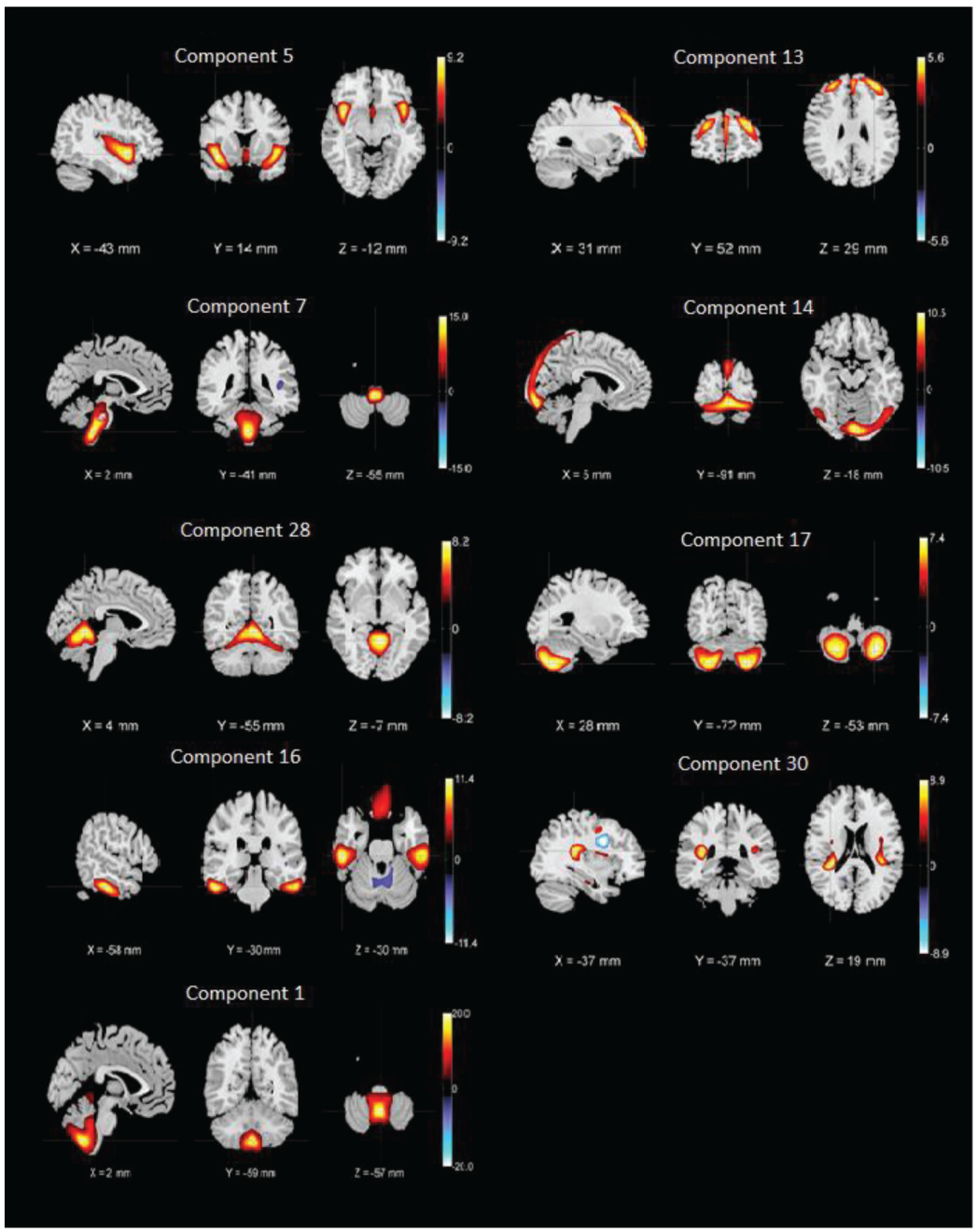
Nine SBM components whose loading parameters were included in the analysis. The components shown are those that are discriminative between patients and controls (higher Ct/SZ group effect) in Ref. [20]

Our study involved participants from three different studies FBIRN3 [33], TOP [20] and COBRE [34]. We formed an aggregated dataset consisting of 382 SZ subjects (mean age = 36.4, SD = 11.65, range: 18–64, 274 males/108 females) from nine scanning sites, along with individual symptom scores. PANSS information captures positive and negative symptoms and gauges their relationship to one another and to global psychopathology [35]. The SZ subjects were on antipsychotic medications (either typical, atypical or a combination) and were clinically stable at the time of scanning. The demographic and clinical information about the dataset is provided in Table III. All datasets used the structured clinical interview for diagnosis for DSM-IV or DSM-IV-TR to confirm a diagnosis of SZ or schizoaffective disorder. We have used the same preprocessing pipeline as our previous studies [3, 20]. More information about the datasets is available in the supplemental material (see Appendix 1) of a prior study [20]. We applied the method on all 30 components extracted by ICA from the aggregated dataset. However, we focused on nine group differentiating components that are shown in figure 6. We also included three symptom scores (positive, negative and general) from the PANSS to potentially capture biclusters, including the symptom scores that resulted in a set of 12 distinct features.

**Fig. 7.**
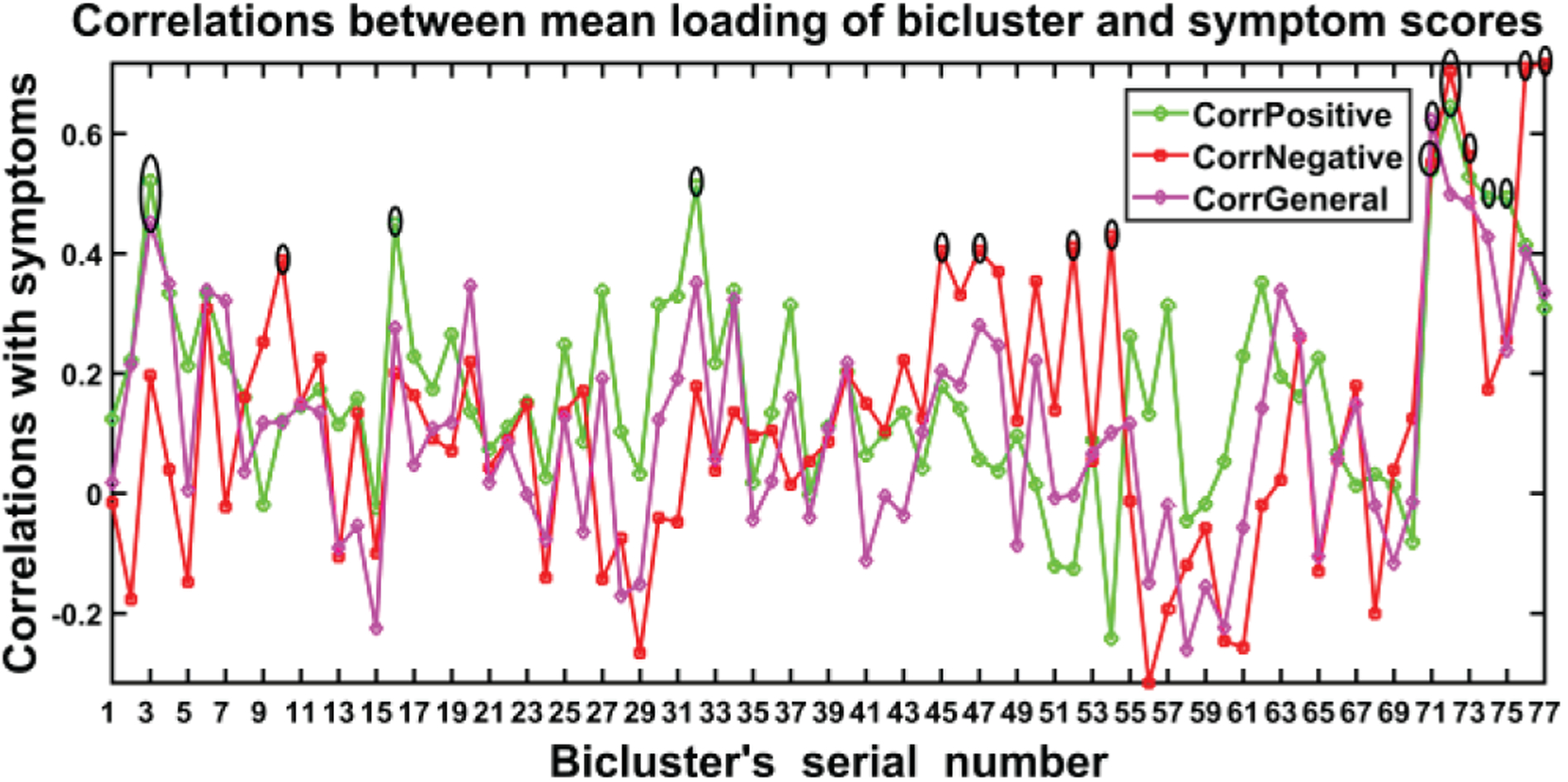
Correlations between the symptom scores and biclusters (77) extracted by N-BiC. Three colors represent correlations with three different symptoms scores and the tall spikes indicate significant correlations which are the point of interest for reasoning the SZ symptoms. The black ellipsoid points are significant with FDR correction for multiple comparisons.

**Fig. 8.**
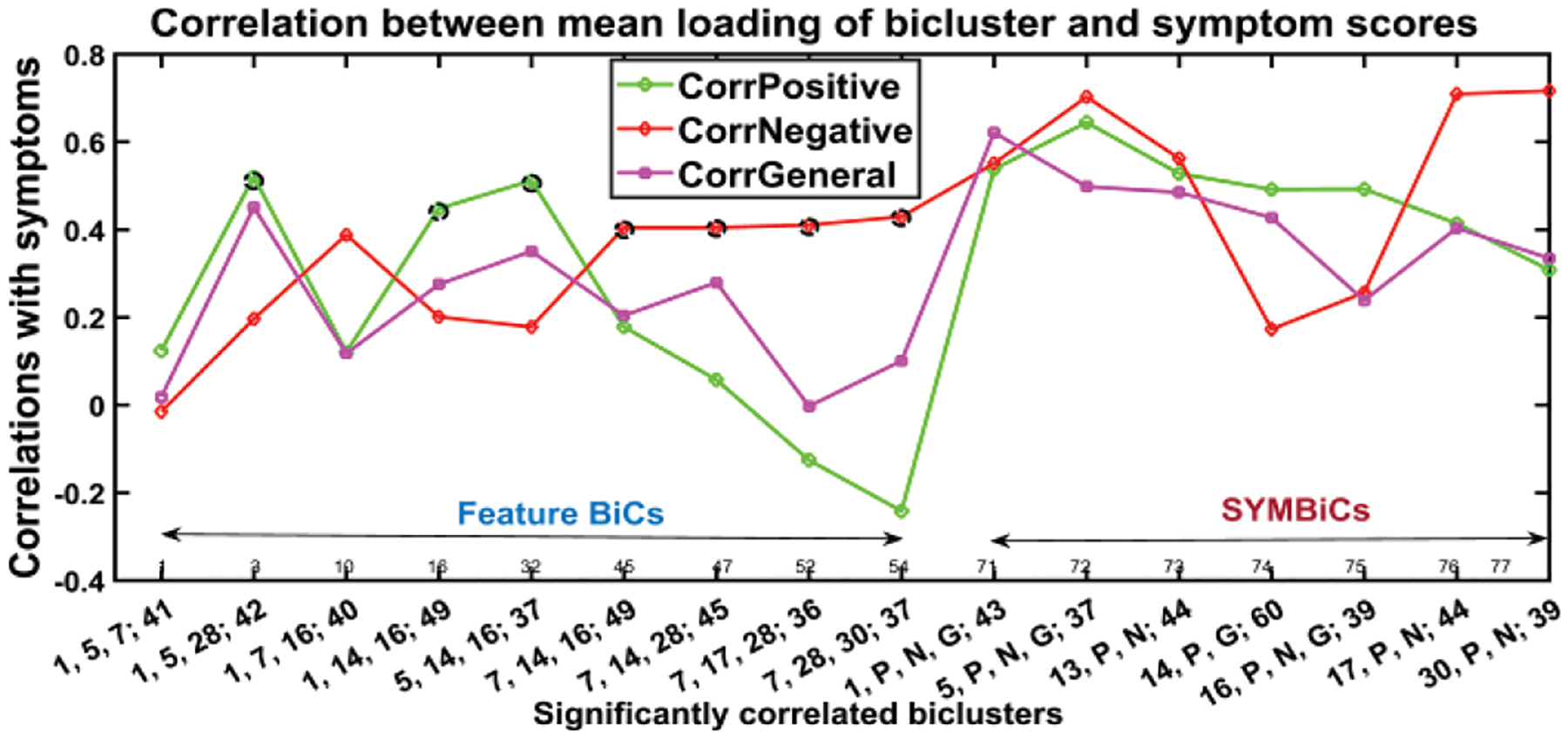
Biclusters significantly correlated with (at least one) symptom score (p-value ≤ 0.05). Included are the biclusters, which have tall spikes in figure 7, and classified the BiCs into two subgroups feature BiCs and SYMBiCs Feature BiCs that include only SBM components and SYMBiCs consist of three symptom scores and 1or 2 SBM components. Significant positive correlations are denoted by black circles; significant negatives are blue circles and the significant SYMBiCs are represented by ellipsoids. (Numbers in x-axis represent components and subjects within that bicluster. The number before the semicolon and separated by commas stand for components and the number after the semicolon represents the number of subjects included in that bicluster.)

Our algorithm performed biclustering for all permutations of the concatenated set of 12 components and initially obtained more than 300 biclusters. After stabilization and validation, this was reduced to 77 informative and stable BiCs (biclusters) as shown in figure 7. Next, we measured correlations between the symptom scores and summarize significantly correlated biclusters in figure 8.

Figure 7 shows the 77 biclusters estimated by N-BiC algorithm primarily. It has several high spikes which are denoted by black lobes. Those peaks represent the high correlation with corresponding symptom scores. The highly correlated biclusters are presented along with their statistical significance (p-values) in Table IV, V, and VI respectively after FDR (false discovery rate) corrections. The asterisk (*) sign with a correlation value is used to denote it as significant. We picked biclusters with a significant p-value and a higher correlation value (≥0.4) with at least one symptom score.

Figure 8 shows the 16 BiCs significantly correlated with symptoms. We used these 16 BiCs from a list of 77 to describe how the correlations change with different factors and visualized the results in a compact way that makes the interpretation easier. Each bicluster has a *p*-value ≤ 0.05 with at least one of the symptom scores in the above figures. The set of biclusters are divided into two subgroups. As we mentioned earlier, nine (BiC 1 – 9) are feature biclusters and seven (BiC 10 – 16) are SYMBiCs. The feature biclusters consist of SBM components only and of these nine biclusters have a high correlation with positive symptoms are found for BiC 2, 4, and 5. These three biclusters are mostly dominated by components 1, 5 and 16 and include inferior semilunar lobule (ISL), superior temporal gyrus (STG) and inferior frontal gyrus (IFG), inferior temporal gyrus (ITG) and fusiform gyrus (FG) respectively. When the biclusters include component 7 (brainstem (BST)) instead of 1, 5 or 16 it is not significantly correlated with positive symptoms (see BiC 6, 7, 8 and 9). Bicluster 9 has a higher correlation with negative symptoms only. The correlation between positive symptoms diminished when component 7 (BST) is included. From Table IV, we can see the statistical significance of those correlations with negative symptoms; most of the *p*-values for those biclusters with negative symptoms are ≤ 0.05. This suggests component 7 (brainstem) is associated with negative symptoms in schizophrenia. Bicluster 3, including 40 subjects and three components, shows low correlation with positive and general symptoms but a higher correlation with negative symptoms (0.39, p = 0.0253). BiC 10, 11, and 13 are SYMBiCs since they include all three symptom scores. BiC 10 and 11 have a high correlation with three symptom scores. This includes components 1 and 5 respectively, which might be a potential biomarker for those symptoms. As mentioned earlier, biclusters 2, 4, 5 have three dominating components: 1 (ISL), 5 (STG & IFG) and 16 (ITG). But SYMBiCs 10 (includes component 1) and 11 (includes component 5) show a higher correlation with negative symptom scores and SYMBiC 14 (includes component 16) shows comparatively higher inclination with positive symptom scores only (although these biclusters include all three symptom scores). The presence of component 16 (inferior temporal gyrus) appears to be the key reason for the biclusters 2, 4 and 5 association with positive symptoms.

**Fig. 9.**
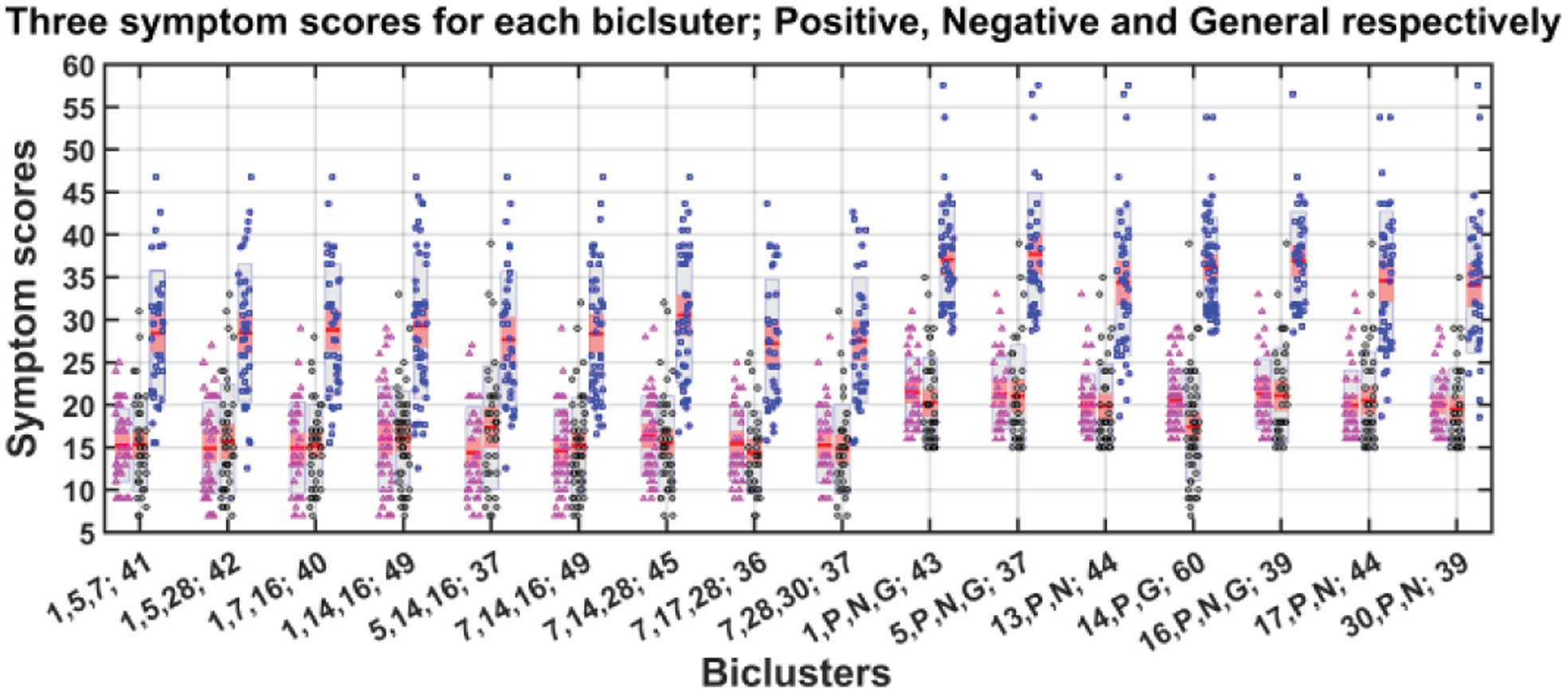
Mean and standard deviation of symptom scores for each bicluster. Each bicluster has three bars representing positive (magenta color dots), negative (black) and general (blue) symptom scores. The dots represent the real data (subject-wise symptom score) and red line in the middle of all bars indicate mean of symptom score. The gray area indicates grouped raw data in mean ± SEM and pink area: mean ± SD.

Figure 9 depicts the mean and standard deviation of symptom scores of each bicluster. Biclusters 2, 4, 5 follow a similar pattern for three symptom scores. The mean and standard deviation for the positive symptom scores of these biclusters are very similar. All the SYMBiCs from BiC 10 to 16 have a higher average of general symptom scores. Those subjects can be identified as a probable subgroup of higher general symptom severity where general symptoms are more severe than the positive and negative.

**Fig. 10.**
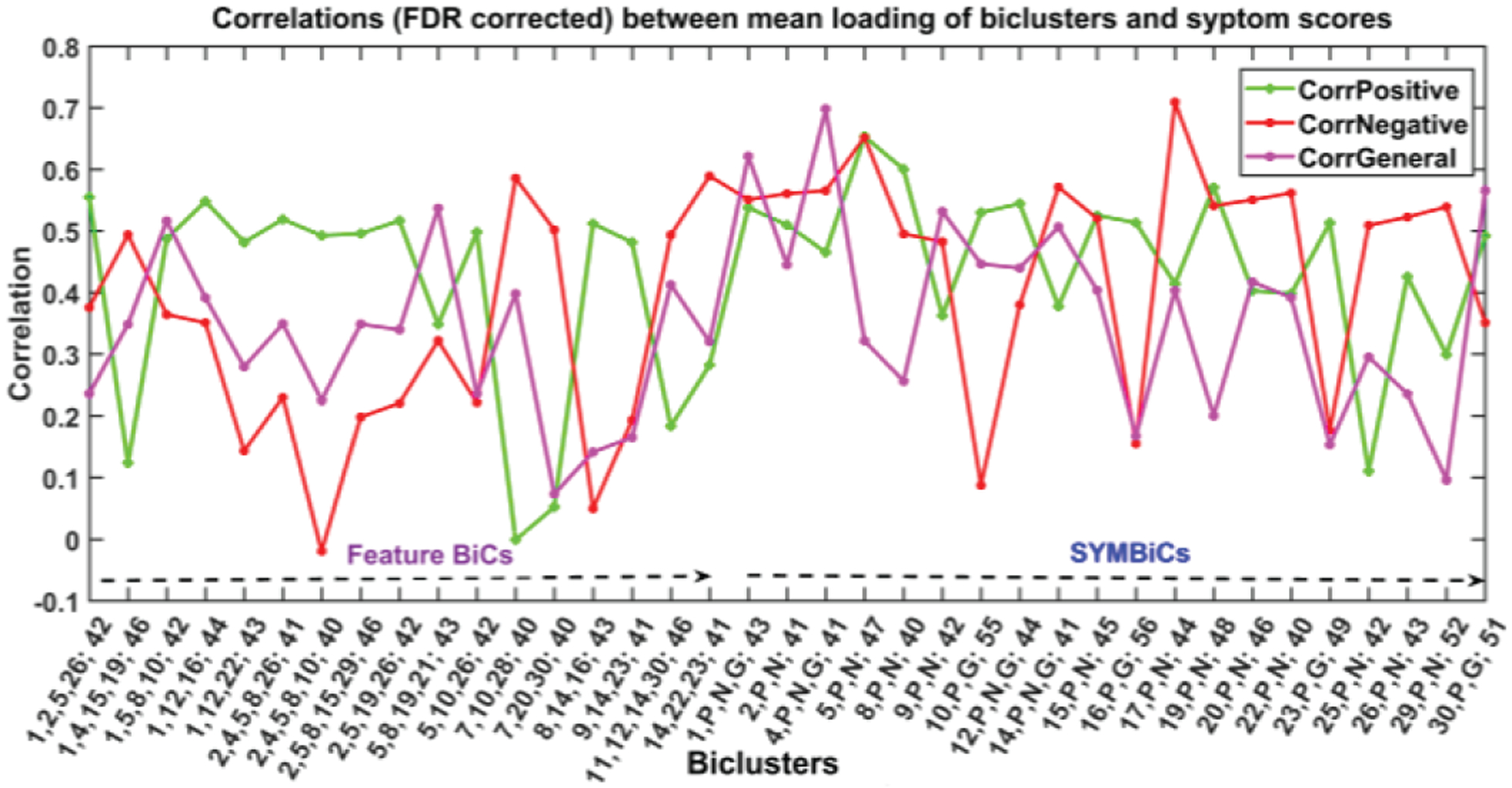
The figure demonstrates results for an extended run of N-BiC for all 30 components and three symptom scores. The biclusters are significantly correlated with (at least one) symptom score (≥ 0.05, FDR corrected). It reports 34 BiCs from a vast set of more than a thousand biclusters primarily collected for an input parameter setup (minComp = 3, minSub = 40, overlap = 20 %). The BiCs are divided into two subgroups feature BiCs and SYMBiCs. Feature BiCs include only SBM components and SYMBiCs consist of three symptom scores and 1 – 2 SBM components. The numbers in xticklabel represent components and subjects included in the corresponding bicluster. (The number before the semicolon and separated by commas stands for components and the number after the semicolon represents the number of subjects included in that bicluster.) Here, P, N, and G stand for positive, negative, and general symptom scores respectively.

We also extended the analysis to include all 30 components and observed consistent outcomes to the earlier results with additional findings. In this run, we set the minimum required subjects (minSub) and components (minComp) in a bicluster to 40 and 3 respectively. The allowed overlap is 20%. In figure 10, we present 37 biclusters that are highly correlated with at least one symptom score (>=0.5, FDR corrected).

This run extracts two times more biclusters of diverse sizes than the earlier run. Since we set minComp = 3, it reports biclusters with the size 3/3+. We divided the set of biclusters into two subgroups, feature BiCs, and SYMBiCs, and here also we observed the domination of components 1, 5, 7, 16 along with newly added components 2, 8, 10, 12, etc. across all feature BiCs. Furthermore, we can see how the biclusters consist of components 1, 2, 5, 16 roughly show a higher correlation with positive symptoms (BiC 3, 4, 5, 6, 7, 8, 9, 11 etc.). Consistently, the association of component 7 (brainstem) shows a higher correlation with negative symptom scores and a lower correlation with positive scores (BiC 12, 13). Additionally, we identified a set of biclusters comprised of components 2, 4, 5 and 8 that show a higher correlation with both positive and negative symptom scores. This subset of components is worth studying for characterizing schizophrenia in these subgroups of subjects. In SYMBiCs, we found a distinct individual association of SBM components with symptom scores. Moreover, this extended run also shows a similar set of insignificant components (e.g., 3, 6, 11, 18, and 24) with the symptom scores. Overall, running the analysis for all 30 components is consistent with but extends our initial into a more focused analysis.

## IV. Discussion and Parameter selection

The study demonstrates a novel method for biclustering neuroimaging data. The algorithm is applicable to various MRI data (sMRI, fMRI, diffusion etc.) and suitable for smaller sample size (n = 20–30). It is a purely data-driven method for extracting homogenous patterns of an individual’s expression level (i.e., loading value) to a subset of distinct SBM components. An SBM decomposition technique (i.e., ICA) evaluates a prespecified number of sources (components) by decomposing the subject’s MRI images and provides them to N-BiC, which eventually discovers the biclusters embedded in a given dataset disregarding their size. In other words, the algorithm is unbiased to the size of the dataset, but it requires careful tuning of size parameters to avoid observing insignificant biclusters. The results present two subgroups of biclusters; feature BiCs and SYMBiCs where BiC 1 to 9 are feature BiCs and BiC 10 to 16 are SYMBiCs. The SYMBiCs are a special type of bicluster with an evident strong association of specific SBM component with PANSS scores. These reports address several subgroups of subjects homogenous to certain SBM components clustered with 2/3 symptom scores; hence, cab = n may be used as the indicator for characterizing the different types of schizophrenia. In addition, the approach can potentially explore the variability of the dataset in a more granular manner than other methods in the literature [3, 36]. Arnedo et al. introduced a general factorization method (GFM) that uses non-negative matrix factorization (NMF) for creating the submatrices (clusters). It is a voxel level clustering that tries to identify a homogenous subgroup of subjects sharing similar patterns (a subset of voxels) by minimizing the objective function fractional anisotropy (FA) (maximizing homogeneity). Similarly, N-BiC clusters the SBM components maximizing similarity in loading parameters and extracts subgroup of subjects covary loading coefficient across a subset of components. Both methods handle overlap between biclusters and control redundancy in the results by using the similarity index (F-statistics, hypergeometric distribution etc.). However, FNMF requires a factorization parameter k (corresponds to a maximum number of biclusters) that determines how the data matrix will be decomposed, and it can hold a maximum value √n where n is the number of subjects. This restricts the capacity of the method in terms of a number of extractable biclusters [36]. In contrast, our method uses some minimum size/shape parameters to shift through the data without restricting the natural capacity of the algorithm. Moreover, the reference method constructs a brain region by clustering voxel by voxel depending on FA reduction across a subset of subjects and our method clusters networks (SBM maps) based on the loading coefficient of corresponding subjects towards generating a subset of networks. Both studies indicate distinct regions of the brain for characterizing schizophrenia within the reported subgroups. Our approach is also more robust than the method described in Gupta et al. in term of size, variability, prior information about the dataset [3]. In this project, we applied our approach to structural MRI data of schizophrenia patients. Results reveal the existence of many biclusters, of which 77 were found to be stable and 16 showed a significant relationship with symptom scores. The behavioral relevance of these biclusters indicates the association of discrete brain regions toward a specific type of behavior in schizophrenia. We categorized the set of significant biclusters into two major subgroups. The first nine biclusters are featured BiCs (includes MRI features only) and the biclusters from 10 to 16 are SYMBiCs (includes symptom scores). Three biclusters (2, 4, and 5) were dominated by components 1 (ISL), 5 (STG and IFG) and 16 (ITG), showing a significant relationship with the positive symptom scores. In other words, abnormalities in these three anatomical regions had distortion in those subgroups of SZ subjects. These components could be used as potential subgroups/subtypes or biomarkers for schizophrenia in the later study. We introduced the SYMBiC – bicluster, which includes three symptom scores and at least one brain component. BiC 10, 12 and 14 are three important subgroups of subjects that have an important relationship with the PANSS score. The correlation with symptom scores has established component 16 (inferior temporal gyrus) as persistently associated with positive symptoms in schizophrenia. There also are biclusters that highly correlated with negative symptoms. Biclusters 3, 6, 7, 8, and 9 have a high correlation with negative symptoms, but a lower correlation with the other two symptom scores.

The common component among those listed biclusters is 7 (brainstem), suggesting it plays a key role in this relationship. These associations of components 16 (ITG) and 7 (BST) with two distinct sets of biclusters are compelling since each of the components are replicated across several distinct subgroups of subjects and potentially control the group’s behavior. These may suggest multiple complex relationships between the subgroups of subjects and the components. The biclusters which show meaningful relationships with any of the symptoms may also provide evidence of possible subgroups/subtypes. The N-BiC approach thus may serve as a way of identifying a possible biomarker of illness and provides an important link between symptom scores and biological features and should be replicated in future studies.

In our algorithm, there a few parameters and thresholds that need to be set before running the processing. The parameter setup of our algorithm is mostly data specific and partially dependent on user preferences. We have parameters for the size of the bicluster (N, K), the percentage of overlap between two biclusters (O) and the preference for the component sorting method selection (M). Since the method runs for all permutations of a set of given components, the thresholds fTH1 and fTH2 are used to check the similarity level between a newly discovered BiC and the biclusters that are already reported. By tuning the overlap percentage parameter O, we can easily calibrate through the number of biclusters as well as the replication in the results. The more overlap allowed the lower the thresholds values, so we will get more replicated/overlapped biclusters. By decomposing a dataset into more precise subgroupings, investigators can allow less overlap between two biclusters. We are basically suggesting an algorithm to treat a subgroup (which has a greater portion overlapped with an earlier bicluster) as a different bicluster, rather than merging it with the earlier one. Eventually, it spins up the number of biclusters and thus the overall number of duplicates in the results set. This comprehensive way of traversing the search space is obviously time-consuming. The driving function of this algorithm is DFS, which uses a backtracking policy for reporting all possible subsets of a given dataset. The maximum time complexity of this modified DFS step is O(2^n^) for a given set of length n. Apparently, the order in which DFS processes the set elements (components) does matter since it picks the first element with a probability 1 to evaluate intersection with others. We used a permutation step to generate all possible order of components and ran the processing for each one. Finally, we reported a convergent set of biclusters across all these permuted runs. For this robustness to be invariant to a starting point/seed, the approach accumulates the count of time complexity by O(n!) asymptotically. The cumulative time complexity of the algorithm is an exponential function of input size, O(n!). However, the algorithm ensures the traversal of every single combination of target variables (components) in the search space. This is the cost of robustness for performing an exhaustive search, though future optimizations are possible.

This kind of selection might be necessary for any post hoc analysis of biclustering results. By selecting a lower value for the allowed overlap parameter, we are being aggressive about the overlap. There is a chance of losing some significant biclusters, but it would increase the overall quality of biclusters by removing the duplicated biclusters too. Here, duplicate means a bicluster that has common subjects and features with other already enlisted biclusters. There is a tradeoff between the quality of biclusters and the broadness of discovery. Hence, a balance should be identified for this parameter before running the algorithm. The minimum size parameter (S, K) is also important for extracting meaningful biclusters. If we allow smaller BICs, this might result in a larger number of biclusters that may have less meaning and be difficult to interpret. For example, if we are running correlation or regression or coherence on a set of resulting biclusters, we must have a moderate number of subjects and features in our biclusters to minimize correcting for multiple statistical tests. Moreover, if the parameters are too small, then the algorithm would extract lots of smaller biclusters containing significantly less information. On the other hand, if we use a large parameter, we might end up with no biclusters or very few biclusters. We ran our data for different combinations of parameters to come up with an optimal configuration. We ran data for (S, K, O) = (20, 2, 20), (20, 3, 20), (20, 3, 33) (30, 3, 20), (35, 3, 20), (35, 3, 35) etc. For the pairwise checking, K = 2 performed well at extracting significantly correlated (with symptoms) biclusters and a fair number of BiCs with interpretable size. Since in most cases we used a minimum number of components, ≤ 3, it is important to carefully select the overlap parameter. For example, if we are set K ≤ 3 and O ≤ 20, then we are allowing 20% of 3 which is ≤ 1. If we select a higher value for the minimum number of subject parameters, then this results in an insufficient number of components that directly affect the total number of extracted biclusters. By balancing these extremes, we determined that (3, 35, 35) is a convenient configuration for our dataset. This provides a moderate number of interpretable biclusters that offer satisfactory insights about the dataset.

## V. Conclusion

The aim of this project was to develop a data-driven biclustering approach for neuroimaging data that can analyze multiple pairs of features (component loading parameters in our case). The approach can be applied to both patients and healthy controls data and is more robust than any other previously reported studies, especially considering that N-BiC is a fully exhaustive model for exploring all possible combinations of data variables without any prior information and a specified direction. Additionally, the algorithm can process an arbitrary number of subjects and features. Further, it can uncover intrinsic data substructures unbiasedly by not using any information about the expected number of biclusters as was addressed in previous biclustering studies in the literature [3, 36]. This two-dimensional subgrouping of individuals and covarying brain components provides substantial advantages over conventional clustering approaches by exploring homogeneity across a subset of features unlike all over the feature space in classical clustering [37, 38]. This identifies interesting patterns replicated among a subgroup of individuals. Using behavioral data as a neural feature (SYMBiC), the approach automatically clusters them with ICA components that are strongly evident in the association with a specific symptom in schizophrenia. The SYMBiCs suggest several discrete brain regions; irregularity in those areas can potentially lead to distinct classes of schizophrenia. N-BiC also allows the user to calibrate various parameters to explore different aspects of a dataset.

In the future, we plan to run this approach on healthy control data for comparison with the current results and also for incorporation within a classification approach to see how it differentiates between a new dataset of patients and healthy controls. A potential direction would be to incorporate some prior information (such as clinical scores, probabilistic selection of parameters and thresholds) to evaluate their roles on the grouping in terms of improving the performance of the algorithm.

## Figures and Tables

**TABLE I:** Ground truth biclusters embedded in the data matrix (created in simulation-1) for measuring the mean square residue and consensus score to check the performance of components sorting methods

BiC	Subjects	Components
1	[278, 342, 118, 261, 12, 378, 313, 331, 295, 318, 163, 53, 64, 327, 133, 218, 330, 380, 185, 28, 209, 79, 120, 391, 67, 311, 137, 190, 41, 25, 345, 29, 354, 158, 177, 141, 13, 291]	[2,9,3]
2	[271, 195, 325, 31, 12, 174, 58, 324, 1, 39, 169, 95, 383, 149, 197, 108, 231, 209, 392, 265, 339, 35, 170, 17, 241, 293, 274, 25, 27, 123, 140, 291, 43, 101, 176]	[1,4,5]
3	[125, 367, 264, 71, 259, 15, 314, 1, 154, 269, 16, 174, 70, 234, 77, 145, 353, 10, 273, 114, 329, 118, 157, 381, 335, 357, 374, 52, 140, 286, 251, 334, 89, 128, 88, 12, 237]	[6,7,8]
4	[46, 29, 233, 5, 338, 27, 194, 65, 169, 346, 263, 385, 159, 165, 312, 361, 268, 69, 128, 109, 93, 281, 124, 390, 345, 248, 352, 123, 251, 254, 121, 234, 44, 320, 351, 260, 23, 376, 24, 100]	[2,4,6]

**TABLE II:** List of ground truth biclusters (simulation-2) for testing the N-BiC approach

Subjects	Components
[1, 2, 3, 4, 6, 7, 21, 27, 29, 31, 35, 38, 40]	[1,8, 3]
[5, 13, 17, 18, 19, 20, 23, 24, 26, 28, 30, 33, 34]	[2, 4, 5]
[8, 9, 10, 11, 12, 14, 15, 16, 22, 25, 32, 36, 39]	[6, 7, 9]

**TABLE III:** Demographic and clinical information of aggregated dataset by study

Dataset	Sample size	Male/female	Age (mean ± SD)	Sites	PANSS positive mean	PANSS negative mean ± SD	PANSS general mean ± SD	% Reporting Duration of illenss (DOI)
FBIRN3	179	136/43	39.22 ± 11.6	7	15.55 ± 5.11	14.44 ± 5.50	27.90 ± 7.26	98.30
TOP	128	76/52	31.80 ± 08.9	1	14.60 ± 5.23	15.0 ± 6.78	27.80 ± 8.15	97.54
COBRE	75	62/13	37.56 ± 13.5	1	15.42 ± 4.86	14.76 ± 4.94	27.90 ± 8.63	98.70

**TABLE IV:** Biclusters correlation with positive symptom scores with their FDR adjusted *p*-values. The threshold used for significance is 0.05. The *p*-values presented here are after taking 4 decimal places only.

BiC Id	Number of Subjects	Components	Positive symptom		
			Correlation	*P*-Value	Adjusted *p*-value for FDR
**1**	41	1, 5, 7	0.1242	0.4390	0.5225
**2**	42	1, 5, 28	0.5210*	0.0004	0.0017
**3**	40	1, 7, 16	0.1207	0.4579	0.5225
**4**	49	1, 14, 16	0.4483*	0.0012	0.0039
**5**	37	5, 14, 16	0.5123*	0.0011	0.0039
**6**	49	16, 7, 14	0.1788	0.2188	0.2763
**7**	45	7, 14, 28	0.0576	0.7068	0.7539
**8**	36	7, 17, 28	−0.1252	0.4665	0.5225
**9**	37	7, 28, 30	−0.2415	0.1497	0.2178
**10**	43	1, P, N, G	0.5380*	0.0001	0.0010
**11**	37	5, P, N, G	0.6448*	0.0000	0.0001
**12**	44	13, P, N	0.5281	0.0002	0.0011
**13**	60	14, P, N	0.4921	0.0000	0.0004
**14**	39	16, P, N, G	0.4929*	0.0014	0.0042
**15**	44	17, P, N	0.4145	0.0051	0.0123
**16**	39	30, P, N	0.3085	0.0559	0.0926

**TABLE V:** Biclusters correlation with negative symptom scores with their FDR adjusted *p*-values. The threshold used for significance is 0.05. The *p*-values presented here are after taking 4 decimal places only.

BiC Id	Number of Subjects	Components	Negative symptom		
			Correlation	*P*-Value	Adjusted *p*-value for FDR
**1**	41	1, 5, 7	−0.0147	0.9273	0.9470
**2**	42	1, 5, 28	0.1969	0.2112	0.2740
**3**	40	1, 7, 16	0.3886*	0.0131	0.0253
**4**	49	1, 14, 16	0.2014	0.1650	0.2263
**5**	37	5, 14, 16	0.1785	0.2902	0.3572
**6**	49	16, 7, 14	0.4048*	0.0039	0.0098
**7**	45	7, 14, 28	0.4050*	0.0057	0.0132
**8**	36	7, 17, 28	0.4108*	0.0128	0.0253
**9**	37	7, 28, 30	0.4300*	0.0078	0.0164
**10**	43	1, P, N, G	0.5512*	0.0001	0.0007
**11**	37	5, P, N, G	0.7030	0.0000	0.0000
**12**	44	13, P, N	0.5626*	0.0000	0.0004
**13**	60	14, P, N	0.1734	0.1851	0.2468
**14**	39	16, P, N, G	0.2568	0.1144	0.1771
**15**	44	17, P, N	0.7094*	0.0000	0.0000
**16**	39	30, P, N	0.7168*	0.0000	0.0000

**TABLE VI:** Biclusters correlation with general symptom scores with their FDR adjusted *p-*values. The threshold used for significance is 0.05. The *p*-values presented here are after taking 4 decimal places only.

BiC Id	Number of Subjects	Components	General symptom		
			Correlation	*P*-Value	Adjusted *p*-value for FDR
**1**	41	1, 5, 7	0.0184	0.9088	0.947
**2**	42	1, 5, 28	0.4513*	0.0026	0.0071
**3**	40	1, 7, 16	0.1180	0.4681	0.5225
**4**	49	1, 14, 16	0.2757	0.0551	0.0926
**5**	37	5, 14, 16	0.3511	0.0330	0.0610
**6**	49	16, 7, 14	0.2044	0.1587	0.2240
**7**	45	7, 14, 28	0.2799	0.0625	0.1000
**8**	36	7, 17, 28	−0.0025	0.9881	0.9881
**9**	37	7, 28, 30	0.1012	0.5510	0.6011
**10**	43	1, P, N, G	0.6215*	0.0000	0.0001
**11**	37	5, P, N, G	0.4986*	0.0016	0.0047
**12**	44	13, P, N	0.4852*	0.0008	0.0031
**13**	60	14, P, N	0.4275*	0.0006	0.0026
**14**	39	16, P, N, G	0.2390	0.1428	0.2142
**15**	44	17, P, N	0.4039*	0.0065	0.0142
**16**	39	30, P, N	0.3351	0.0370	0.0658
